# Surfacing Technology Routines While Studying Videoconferencing Among Older Adults with Cognitive Concerns

**DOI:** 10.1145/3706598.3714207

**Published:** 2025

**Authors:** Ruipu Hu, Amanda Lazar

**Affiliations:** College of Information, University of Maryland, College Park, Maryland, USA; College of Information, University of Maryland, College Park, Maryland, USA

**Keywords:** Videoconferencing, Older Adults, Cognitive Concerns, Design for Aging, Accessibility

## Abstract

HCI research increasingly focuses on everyday life to inform technology design for older adults. Routines, a key aspect of everyday life, have been studied to contextualize technology use. Our work brings attention to understanding of routines around videoconferencing technology among older adults with cognitive concerns. We conducted a week-long study involving observations, interviews, and a modified diary study with six older adults with cognitive concerns who videoconference at least once a week. Our analysis revealed how routines helped people adapt to videoconferencing constraints, how participants navigated disruptions to their videoconferencing routines, and the kinds of routines that were more challenging to manage when faced disruptions. In the discussion, we describe why routines are particularly important to study and support for people with cognitive concerns, the importance of studying older adults’ routines to support technology use in HCI, and methods that can enrich HCI research by uncovering insights into routines.

## 1 Introduction

Videoconferencing has been an application of great interest for researchers studying technologies for older adults. The pandemic brought new uses and users to videoconferencing [8, 76].For instance, telemedicine has made healthcare consultations more accessible for older adults, especially those with mobility constraints or limited access to in-person care [35, 43, 81]. The opportunity for widespread usage persists as professional, cultural, health, and familial interactions continue or are increasingly available via videoconference. Our interest in working with people with cognitive concerns on this topic stems from the need we recognize for ensuring videoconferencing is cognitively accessible given its increasing use.

As technologies become more widely used, people may develop routines around them. Routines, often described as a repeated behavior that involves a momentary task requiring little conscious effort [5], such as eating breakfast or drinking water, provide structure to daily activities. Routines have been studied in various fields to understand how repeated behaviors help people respond to lifestyle changes [3, 5]. For example, a bedtime routine is often associated with improved sleep [5]. Computing researchers have also noted the benefit that can be gained by studying and centering routines, with one paper referring to them as “the very glue of everyday life” (p. 399, [69]).

HCI researchers working with older adults have similarly noted routines as pointing to promising design opportunities [15, 31, 37]. For example, Brereton et al. designed around an existing “comfort routine” involving boiling water to make tea [15]. Their project augmented this routine with a messaging system that supported older adults in staying connected with others [15]. This past work has primarily sought to understand how new technologies can be designed to support existing routines of older adults. Our aim in this paper, in contrast, is to understand existing routines – and specifically, existing routines that support *existing technology use.* This interest is driven by our realization that, as more technologies become habituated [14] in the everyday life of older adults, studying practices that are rooted in everyday life and developed by older people themselves can inform better technology design.

Despite their repetitive nature, people are often unaware of the specific details of their routines, as these behaviors become automatic through regular practice [3, 5]. Therefore, over the course of a week, we utilized a number of methods to surface routines which included observations, interviews, and a modified diary study. We enrolled six older people with cognitive concerns who videoconferenced at least once a week. Our analysis focuses on the following research questions:

- **RQ1:** How do routines support the use of videoconferencing technology for people with cognitive concerns?
- **RQ2:** How do participants respond to the disruption of these routines?

We found that participants developed videoconferencing routines to stay comfortable, connected, and focused in the face of the unique constraints of videoconferencing, such as needing to stay in one place for a long time or the extra stimulus that comes from being in one’s own home environment while on a video call. We also found that participants managed disruptions to routines around videoconferencing by accepting them, removing them, negotiating them, or developing new routines that worked around them. Finally, we found kinds of routines that were more challenging to manage when faced disruptions. This included when videoconference access routines did not work, when there were no alternative ways to reach the communication partner, and when new cognitive changes challenged previously effective routines.

This paper makes three contributions. First, we build on earlier work that argues the importance of studying older adults’ technology routines [15, 31, 37]. Second, we offer specific insights into the utility of supporting (and not disrupting) the routines of people with cognitive concerns. Third, we offer methodological considerations for surfacing information about routines in participants’ homes.

## 2 Related Work

Below, we describe research on routines in HCI, particularly with older adults, research on videoconferencing with older adults, and research on cognitive concerns and videoconferencing.

### 2.1 Understanding Everyday Lives and Routines

Understanding people’s everyday lives involves examining the behaviors and practices that shape their interactions with the world. Among these, repetitive and recurring behaviors play a crucial role, as these can offer insight into how individuals adapt to their environments and maintain structure in their everyday lives [69]. Scholars across disciplines have long been interested in how such behaviors form, evolve, and influence broader contexts like health [5, 19, 58], technology use [64], and social interaction [51]. One kind of repetitive and recurring behavior is a routine, or a repeated behavior involving a momentary time commitment task that requires little conscious thought [5].

Research in HCI exploring routines often stems from understanding people’s everyday life contexts. A large body of work has sought to understand how people’s daily lives can be supported by technology with routines as the underlying theme, ranging from context-aware computing to embodied interaction [22, 25, 26, 73]. These works recognize that routines are essential in designing effective technological interventions for people’s everyday life contexts. Context-awareness in computing, for instance, emphasizes the ability of systems to sense and adapt to users’ changing contexts in real time [22]. Abowd and Mynatt [1] sought to design systems that seamlessly integrate into people’s lives with the new ideology of “everyday computing” in order to not disrupt people’s existing routines by providing continuous interaction between humans and computers. Similarly, Oulasvirta et al. explored how routines can influence smartphone uses [82]. Along this line, embodied interaction investigates how physical and digital experiences intertwine in the user’s environment, considering routines as situated actions within the context [25]. This type of investigation has been shown to support interventions that enhance daily routines without requiring additional user input outside of the behaviors done as part of a routine.

Researchers working with older adults have also recognized the opportunities that routines offer for design. Some studies have focused on understanding and augmenting specific kinds of routines. By identifying the key objects and activities that people engage with regularly, researchers have designed interventions that build upon existing routines to support their purposes [15, 31, 37, 63]. For instance, Stawarz et al. developed a medical reminder system aimed at integrating into the routine of taking medication to support this essential health-related behavior without introducing significant changes to users’ established habits [63]. Brereton et al. [15] explored how augmenting a seemingly simple routine – boiling a kettle to make tea – could support social connection. These examples of designing with routines highlight the potential of novel technology to fit into and enhance existing routines, thereby promoting health, well-being, and social connection.

Other researchers have studied the processes through which technology use becomes a part of a routine. As part of their findings on the long-term use of voice assistants by older adults, Upadhyay et al. describe routine use (e.g., for entertainment and socialization), linking the development of these routines to a training process that participants had gone through [71]. Ambe et al. demonstrate how dyads collaboratively habituated to a new design, describing factors including aesthetics and the use of embodied skills alongside routines as key to sustained use [31]. While these studies have provided valuable insights into designing technology for routines and everyday contexts, they have involved the deployment of a novel technology. In our study, we examine the routines that older adults with cognitive concerns have already developed around videoconferencing.

### 2.2 Evolving Roles of Videoconferencing for Older Adults

Videoconferencing is a well-established technology that dates back to the late 20th century, initially developed for group work such as conducting remote meetings and conferences [17, 27]. However, it took on new importance during the COVID-19 pandemic, when its role expanded, including for older adults [36, 56, 61, 75, 83, 84]. During this period, videoconferencing primarily became a tool for mitigating the physical isolation imposed by pandemic-related safety measures, enabling populations including older adults to maintain essential social connections despite restrictions [70, 85]. Videoconferencing was also adopted into large community and institutional settings to provide services to older adults [36, 56].

Research on videoconferencing for older adults has primarily evaluated its potential in addressing social isolation and fostering social connectedness [7, 18, 44, 70, 74, 86]. For instance, Tsai et al. demonstrated how a videoconferencing intervention program improved nursing home residents’ social support, loneliness, and depressive status [70]. More recently, research has expanded beyond the social utility of videoconferencing, highlighting a broader range of meaningful uses [2, 4, 28–30, 75]. For instance, videoconferencing technology has come up as a way for older adults to advance social and civic agendas [78] and participate in spiritual and religious events [29].

Despite these benefits, videoconferencing can present challenges that affect users of all ages. Several studies have highlighted that users may not fully engage with the range of videoconferencing features due to usability barriers and a lack of tailored support [6, 67, 87]. Challenges with videoconferencing that have been documented include physical and mental fatigue during extended use [6, 88], difficulties with managing distractions in home environments [87], and a diminished sense of connectedness [67]. These challenges can be pronounced for older adults. Rather than designing new technologies to address these issues which might further exacerbate issues such as the digital divide [60, 89], there is room to explore alternative approaches. In this paper, we examine the role of routines in managing challenges such as those discussed in prior work.

### 2.3 Cognitive Concerns and Videoconferencing

Our project looks into videoconferencing with a focus on older adults with cognitive concerns, who likely face additional barriers to using technologies such as videoconferencing. We use the term cognitive concerns in this paper as a way to account for older people with or without any medical diagnoses who face challenges due to their cognition or memory. We use this term to include three states typically seen on a trajectory of cognitive decline [45]: subjective cognitive decline (SCD)^1^, mild cognitive impairment (MCI)^2^, and dementia^3^. Much current research seeks to intervene in this trajectory by focusing on reduction and early intervention, which relies in part on understanding how people with early cognitive concerns can be supported in maintaining existing functioning by sustaining their current everyday activities [9, 32, 55, 57]. Some of the challenges people with initial cognitive concerns face are associated with difficulties with everyday life tasks involving meta-cognition like planning the next steps, initiating responses to achieve the plan, maintaining organizational material like binder, money [57]. These are all skills that might be called upon to use technologies successfully, including videoconferencing.

Recent research has examined the potential of videoconferencing technology for cognitive testing and clinical assessment [35, 43, 81]. Another study in HCI researched the ways that people with cognitive concerns were currently using videoconferencing [29], finding that participants attended a wide range of activities through videoconferencing, many of which did not necessarily link to cognitive support.

An application of videoconferencing that is being studied in HCI is in supporting remote social programs for older adults with mild cognitive impairment (MCI) and dementia. One study utilized Zoom videoconferencing as a part of their empowerment-focused programming for people with MCI [49]. Another compared and contrasted in-person and virtual social programming offered to people with dementia [91]. These studies have noted the necessity of others assisting people with cognitive impairment in the routine use of the technology. For example, in the transition from an in-person to virtual program, Mynatt et al. discuss how care partners began attending to support people with MCI in accessing weekly content [49]. When care partners were not available, the program drew on staff to support people through “weekly Zoom set up” and other tasks [49]. It is important to understand the social network that supports people with cognitive impairment in using technology successfully, and these studies advance our knowledge in this area. In this paper, we focus on some of the ways that people with cognitive concerns themselves respond to videoconferencing challenges, often successfully.

## 3 Methods

In this paper, we analyze data from a study that we conducted employing interviews, observations, and a modified design study with six participants. We conducted this study to understand how older adults with cognitive concerns engage in video conferencing. Below, we describe the study design, data collection procedures, recruitment strategies, and data analysis process. All procedures were approved by the university’s Institutional Review Board (IRB).

### 3.1 Data Collection

**Figure 1:**
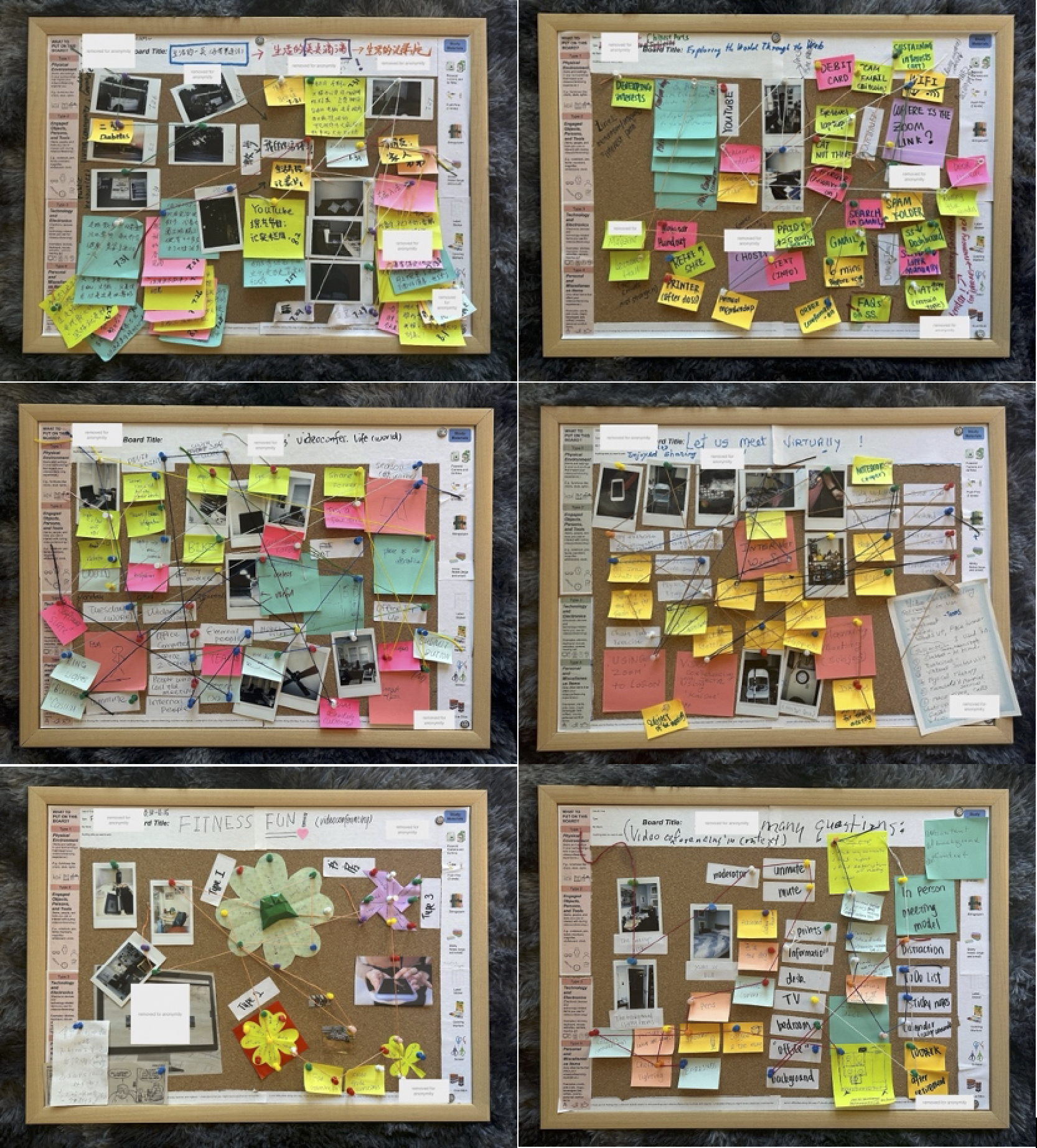
Results of participants’ RAO boards, arranged by rows: first row shows Vivian and Ian; second row features Connor and Lydia; third row includes Nora and Phoebe.

Data collection took place over roughly one week for each participant. We collected data through interviewing participants (21.5 hours in total), observing their videoconferencing spaces (5 hours in total), and completing a modified diary study (which we refer to as a RAO board, as shown in Figure 1 below). Collecting data through these three approaches helped us triangulate data during analysis. We collected data at three time points: during an initial, observation, and reflection session. Over the course of the study, participants filled out their RAO board, which was completed in the final (reflection) session.

#### 3.1.1 Preparatory meeting.

**Figure 2:**
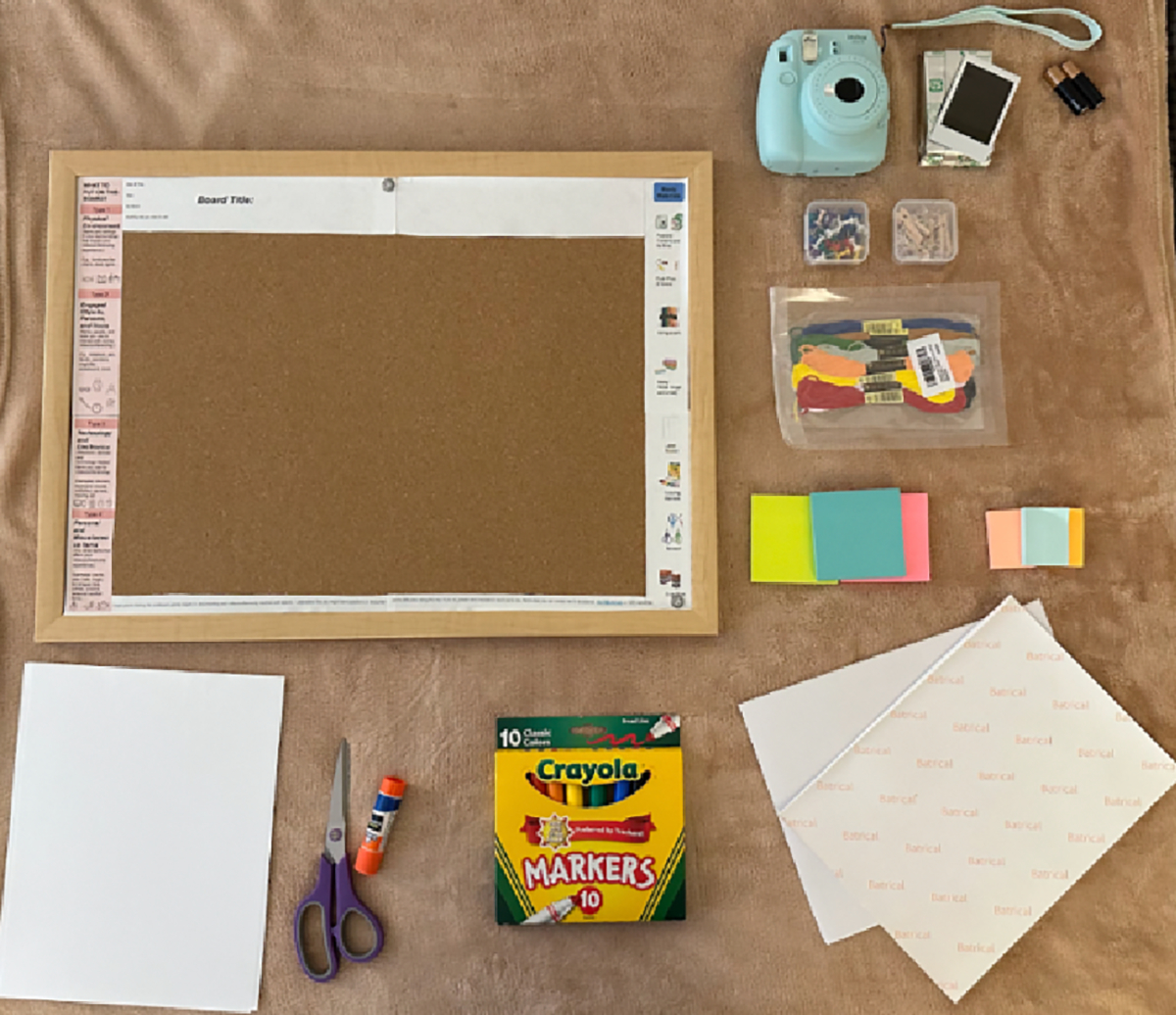
Study materials for the RAO board.

The first meeting between the researcher and participants introduced the study’s goals, activities, and distributed the study materials (RAO board, markers, sticky notes, etc., as shown in Figure 2). We walked participants through the RAO board, giving examples so that we were confident that they would know how to fill it out on their own.

#### 3.1.2 RAO board.

The RAO board (short for Routines and Objects) derives its name from our methodology, which draws inspiration from established HCI practices where identifying routines often starts with examining associated objects [14, 15]. The RAO board was designed to prompt participants to reflect on the granular details of how they were engaging in videoconferencing and to document them in a way that could facilitate further discussion during follow-up sessions.

The RAO board included:

- Board Title: A section for participants to title their board.
- Prompts: A column instructed participants to capture a variety of objects with examples, including from the physical environment, technology, tools, and miscellaneous personal items.
- Study (Design) Materials: Participants were provided with tools such as Polaroid camera, markers, sticky notes, and yarn to document their objects. We provided flexible materials to allow for creativity in how participants represented their routines.
- Contact Information: Contact details for the researcher were included on the board in case participants encountered any difficulties during the study.

Participants filled out the RAO board over the course of the study in the following ways:

- Independent work capturing objects: After the preparatory meeting, participants used the provided study materials to document objects related to their videoconferencing routines over the course of roughly seven days. They were asked to capture objects through taking photographs (with a Polaroid camera), writing notes, sketching, and using other materials, following the instructions provided.
- Researcher check-in: The researcher arrived at participant homes 30 minutes before a scheduled videoconference to review the objects that the participant had documented and address any questions. During the observation of the participants videoconference (see 3.1.3), the researcher observed participants’ interactions with objects and took notes on the setup. After the videoconference, participants and the researcher reviewed the documented objects together to discuss their relevance to videoconferencing use.
- Final Session: In the final session (described more fully in 3.1.4), the researcher and participants worked together to explore the routines associated with the objects that participants and researchers had documented. Participants used yarn to draw connections between objects on the RAO board and discussed how the objects were integrated into their videoconferencing routines. This session provided a platform for participants to reflect on and articulate their routines as the researcher facilitated the exploration of patterns.

#### 3.1.3 Observation.

Participants were selected based on their regular engagement in videoconferencing, and observation sessions were scheduled during a preparatory meeting, allowing participants to choose which session to have the researcher observe. The researcher positioned themselves out of the camera’s view, maintaining a naturalistic environment while observing from a distance. At the sessions, researchers noted any tools, software, or objects people used during the session that participants hadn’t recorded, using the same method as participants (e.g., taking a snapshot with a Polaroid or writing on sticky notes). Photos and videos were also captured during the observations (if taking photos would interfere with the session, they were captured after the videoconference). Additionally, researchers noted items for potential discussion later, for example to ask participants if they wanted to document certain elements or why they chose not to.

#### 3.1.4 Final session.

The final session was used to complete the RAO board as described above. Additionally, several other topics of discussions were raised during this session. First, the researcher asked participants to reflect on their takeaways and perceptions of the board, while also exploring recorded routines in greater detail to uncover hidden or subtle routines. Second, the researchers revisited unresolved questions and observations from the earlier sessions to gather participants’ perspectives on those points. Last, participants were asked to share their thoughts on the method and provide feedback for future design improvements.

### 3.2 Recruitment and Participants

#### 3.2.1 Recruitment.

Inclusion criteria included that participants needed to engage in videoconferencing at least once a week and reside within a specified local area in the United States to facilitate researcher access (with further details omitted for anonymity). Participants had to self-report memory or cognitive concerns to participate in this study, which we describe further in the section below. We also had inclusion criteria that participants were over the age of 50, but all participants were over the age of 60. We selected the age of 50 as the minimum age for participation based on consultation with dementia advocates and a clinician, who explained that this is the decade when age-related cognitive changes can start becoming more noticeable for certain individuals (e.g., people with early onset dementia). All participants received financial compensation ($60) for their time and involvement in the study.

Using convenience sampling, we prioritized diversity in recruitment by seeking to recruit participants who reflected racial, cultural, and living style diversity. Given that a member of our research team speaks Mandarin, we were able to include two participants who preferred to speak in Mandarin during interviews. Both languages of Mandarin and English were also used in other data forms, such as the documentation on the RAO board by participants like Vivian and Nora. The treatment and analysis of multilingual data are explained in the analysis section.

#### 3.2.2 Memory or other cognitive concerns.

Our study required that participants had memory or other cognitive concerns, which we characterized as subjective cognitive decline, mild cognitive impairment (MCI), and mild to moderate dementia. We provided explanations and examples inspired by [79] and [92] to participants. While subjective cognitive decline was self-assessed, to fit in the medical diagnosis categories of MCI or dementia, participants had to have obtained a diagnosis (only one participant was in this category, with a diagnosis of mild cognitive impairment). Participants had to have the capacity to consent to the study in order to participate.

After obtaining informed consent, we gathered additional information about participants’ memory or cognitive concerns during the demographics collection process. These included questions designed to probe deeper into their cognitive conditions, with two MCI-specific questions informed by [32], and five clinical questions developed in consultation with a dementia health professional and based on [93]. These questions aimed to capture a comprehensive understanding of participants’ cognitive concerns across different clinical dimensions that were associated with cognitive concerns, such as amnesia-related issues, aphasia-related concerns, and other factors relevant to cognitive functioning. The screening and demographics material that had to do with cognitive impairment were developed in consultation with a clinician specialist in the area.

#### 3.2.3 Participants demographics.

We report age, gender, race/ethnicity, cognitive concerns, number of year experienced, and examples of cognitive concerns for each participant in Table 1.

### 3.3 Data Analysis

Our analytic approach is based on reflexive thematic analysis [12]. Our initial interest in conducting this project was to understand how we might be able to study videoconferencing routines. This interest in older adults’ technology routines stemmed from the first author’s read of ubiquitous computing literature arguing that technology should better fit into the existing contexts of everyday environments [22, 25, 26, 73], and how that intersected with the literature that both authors took in that emphasizes the importance of routines and meaningful objects in research with older adults [15, 31]. In addition, in past research [49, 59] as well as in our experience working with people with cognitive concerns, we found routines to be a promising inroads to technology use given that they can draw on abilities that persist as other capabilities decline. Further, our analysis was likely shaped by our inclination to see older adults not as inept users of technology but rather as capable and competent agents who must adapt to sometimes unusable or inaccessible design patterns and systems (e.g., in [39]). This way of viewing the world surely shaped the themes we generated, including our analysis demonstrating the purposefulness of routines and participants’ competency in overcoming many kinds of disruptions.

The first step of our analysis involved the first author familiarizing themselves with the data by listening to all audio recordings and verifying transcripts that had been automatically transcribed^4^. The researcher created memos to capture initial thoughts and observations, which were regularly discussed with the last author. Concurrently, the first author generated initial codes for the data. At this stage, and throughout the entire analysis process, both authors met regularly to discuss the emerging analysis. Examples of initial codes generated during this period included “*routine - customizing eyelevel laptop for creating environment he wants*” (where the corresponding data was a routine of placing the computer so that it is eye level), “*routine - avoid distractions*” (corresponding data included a routine of keeping the shades drawn and the doors shut), and “*failed experiment - ring lights*”. The first author proceeded to code all of the collected data, including interviews, observation notes, RAO boards, and other media such as images and videos collected during the study. The first author then inductively grouped codes to generate candidate themes. These included “*Routines Lat Enhance Leir Videoconferencing Experience*” (capturing codes including customizing the eyelevel laptop and avoiding distractions) with the subthemes “*Creating engaging and enjoyable space and ambiance*” and “*Developing environments that minimize disruptions*”; as well as a theme “*when new objects come into their routines*” which included a sub theme “*Reject the new objects*” (which captured the code on the failed ring light experiment). The final step of our process involved iterating on how to define and name these themes, which took place as we wrote and rewrote our findings.

There was a deductive aspect to our approach given that we created a protocol based on and went into analysis with a focus on understanding routines involving videoconferencing. However, we did not come in with any codes or theories that we sought to apply to the data, and were quick to discard assumptions if they did not match the data. Our research focus and research questions narrowed significantly as we conducted the analysis. Therefore, we consider the process largely inductive [13].

### 3.4 Limitations

Our study has several limitations. This includes the small sample size. Our decision to conduct data analysis after recruiting six participants was informed by the depth of engagement with each participant. In addition, we made this decision based on pragmatic considerations [13], including the depth of trust required for people to acknowledge concerns about the stigmatizing topics of cognition and memory and to let us into their homes and guide them through a somewhat involved week-long activity. An additional limitation of our methods is that all data were collected within participants’ homes. While this offers valuable insights into routines in personal environments, it does not capture the full spectrum of contexts where videoconferencing occurs, such as public spaces or workplaces. We recommend future research employ novel methods to explore routines across diverse everyday life contexts for older adults. Also, our study took place over one week. While we did capture some descriptions of how routines developed over time, we are missing understandings of how routines develop that can come from introducing a new technology over several weeks [54] or studying from a long term use perspective [71].

## 4 Findings

In findings section 4.1, we describe how participants used routines to stay comfortable, connected, and focused on face of some of the unique conditions involved in videoconferencing. Section 4.2 details how participants navigated disruptions to their videoconferencing routines. Participants accepted, removed, negotiated, or developed new routines in response to disruptions. While many disruptions to routines were manageable, some were more difficult to overcome. This included when videoconference access routines did not work, when there were not alternative ways to reach the communication partner, and when new cognitive changes challenged routines that had previously been effective for participants. Our findings highlight the purposes of routines and challenges to them, some of which can be overcome by participants and others which persist.

### 4.1 Routines help people adapt to videoconferencing constraints

We found three ways that videoconferencing routines helped participants adapt to unique constraints associated with videoconferencing. Participants developed routines to **stay comfortable** while in one location for a prolonged period of time; to **stay connected** despite the geographic distance from those with whom they were communicating; and to **stay focused** despite the cognitive and sensory demands that videoconferencing brought.

#### 4.1.1 Staying comfortable during a prolonged stay.

Videoconferencing can require that a person stay in one place for longer than they might if they were doing an activity in person^5^. Routines helped participants **stay comfortable despite prolonged stays in one location**. The types of routines that helped participants stay comfortable in our study included *staying hydrated*, *maintaining a comfortable body position*, and *having proper ventilation or air temperature*.

**Figure 3:**
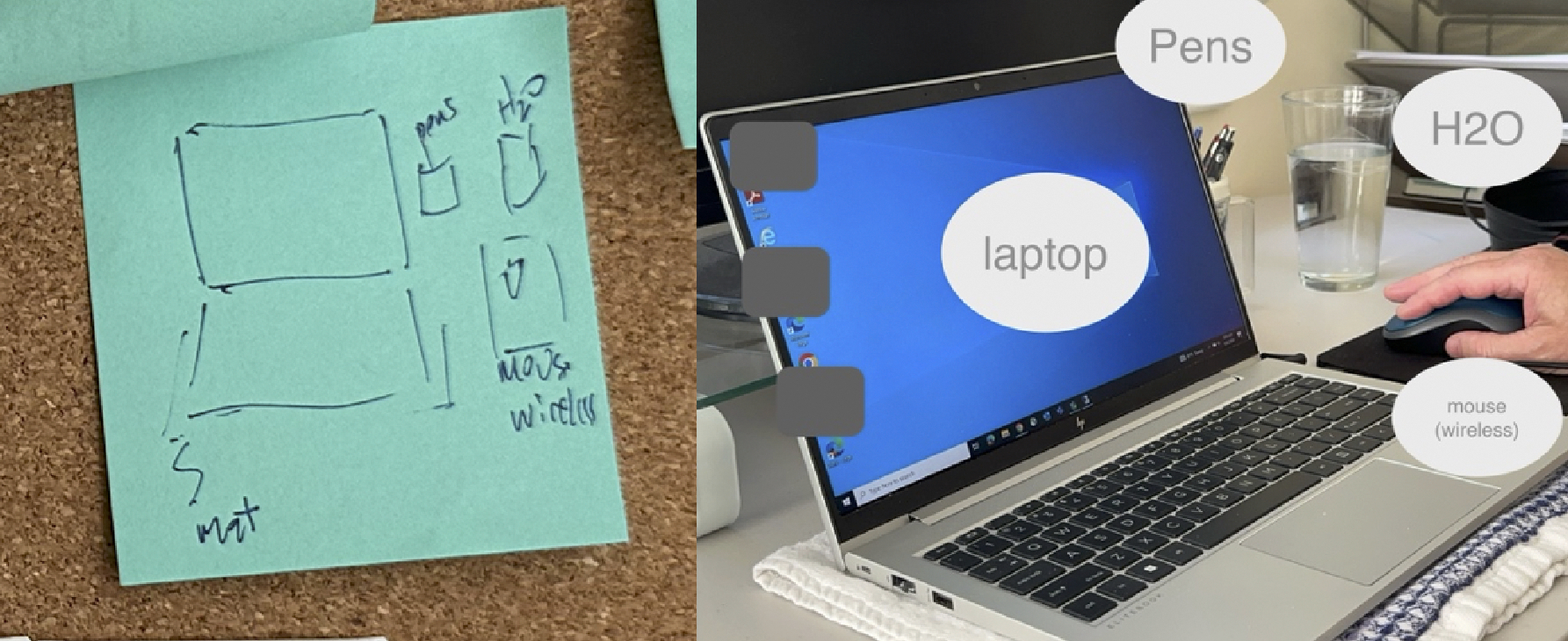
Left: Sketch by Connor of his routine setup on the RAO board. Right: Photo taken by the researcher during observation, reflecting Connor’s routine of including water in his videoconferencing setup before videoconferencing.

Participants had routines to stay hydrated for different kinds of video calls, but this was particularly common for participants engaging in physical activity during a videoconferencing call. The routine here involved, in advance of the videoconference, placing a water bottle in a location that would be within arm’s reach (Connor, Lydia, Nora, Phoebe). This meant that the location of the water varied depending on the type of activity. For video calls that did not require participants to stay fixed around a desk, participants placed their water bottle near the closest surface. For instance, Nora had her water bottle left on the desk as she was standing next to the desk during the videoconference; Lydia placed her water bottle on the coffee table where she can reach by leaning her body and extending her hands. For videoconferences that required people to sit in front of the desk, like the work call Connor was a part of, he put his water bottle near the laptop alongside other crucial objects. This is visible in a sketch Connor created for his RAO board (left image on the Figure 3) which shows his essential setup that includes “H_2_0” in addition to the keyboard, mouse, and computer.

Participants also developed routines to *ensure a comfortable body position*, especially during videoconferences of uncertain or extended duration. These routines often involved choosing the most comfortable furniture in their surroundings as the place to sit or lie on for the duration of the call. For example, Vivian always preferred to sit or lie on the couch to support her back and arms while holding her iPad during videoconferences of uncertain lengths. The choice of seating was based on individual preference: Ian, for instance, opted for ”a comfortable chair that I could sit in for a prolonged period,” as his videoconferences often lasted over an hour. For Connor, the theme of having a comfortable chair also emerged, but through its absence: he described his current seating as “the worst chair ever”.

Participants also developed routines that they engaged in before the call to *maintain proper ventilation*. As our data was collected in the summer, we found much of this involved cooling the space they would be in for the videoconference. If available, participants turned on ceiling fans. If a ceiling fan was not available, they adopted methods such as using a portable fan positioned nearby (Lydia, Phoebe), or opening a window for fresh air (Vivian, Phoebe).

#### 4.1.2 Staying connected across geographical distance.

Videoconferencing brings people “together” in some ways, but can leave or create social and cultural gaps. Examples of this from past work include when videoconferencing brings people together who do not share a time zone or will not see implicit cues in the others’ setting [53]. Routines helped participants in our study **stay connected despite the geographic distance that comes with videoconferencing**. The types of routines that helped participants stay connected in our study included *choosing the language to speak in*, *selecting clothing to match the occasion*, and *following a social order*.

Participants had routines that involved switching to a *shared language* to accommodate the linguistic abilities or preferences of others on the video call. These types of routines helped participants connect linguistically with others on the call. For example, when speaking with her brother, Vivian switched to their shared Shanghai dialect to communicate about topics like seasonal vegetables and the local weather in Shanghai. Vivian had not been able to travel out of the country to see her brother because of the pandemic, and being able to speak in their home dialect about mundane things in her brother’s city made Vivian feel like her brother “is here with me”. Lydia, who spoke English, Hindi, and Gujarati, opted to communicate in their native language when they were speaking to others who shared that language. Lydia explained that this helped them connect more deeply, as using their native language felt like a return to their roots. Altering language can also involve switching from one’s mother tongue to a second language. In Nora’s case, during a videoconference exercise hosted by a local organization where English was the primary language spoken, she used English to participate rather than her native language of Mandarin.

There were also routines of *dressing* in a way that would help people connect socially across distances. This was influenced by how participants perceived formality of the videoconference. Participants adjusted their clothing to be more formal if they considered the meeting to be important or professional. For Phoebe, this was subtle, with her routinely changing which earrings she wore depending on the formality of the videoconferences. Connor, who attended work videoconferences regularly, would routinely wear shorts and t-shirts unless clients or other high-profile individuals were present, in which case he would wear a suit. These outfit changes were different from more functional ones, such as exercise clothing, shoes, and socks for exercise classes (Nora).

We also found participants engaged in routines towards *social order*. This involved having speaking turns regulated by a moderator-like role for some videoconferences. Videoconferencing reduces some of the cues that people rely on for turn-taking, meaning that it can require compensatory work to ensure people are able to speak when they wish to or avoid having one person dominate the conversation [53]. Routines that following a social order led to them being able to connect with one another in a way that was best for the larger group. When the videoconference was moderated by someone like the lecturer in Ian’s webinar or Connor’s boss, participants’ routine involved listening to the moderator, supporting social order, and speaking only when given a cue to do so (e.g., getting called on by the boss). In different social contexts, such as during Lydia and Nora’s videoconference exercise sessions, we found that participants usually follow the routine of not speaking up. However, they also mentioned to the researcher that they could have spoken if necessary. In most cases, the instructor had suggested minimizing speech during the exercise to avoid disrupting the flow, which became a routine they followed even without direct moderation.

#### 4.1.3 Staying focused despite distracting stimuli.

Greater technology use can increase people’s cognitive load [89]. Routines helped participants **stay focused despite the sensory stimuli and cognitive load that arose while videoconferencing in their home space**. Types of routines that helped participants stay focused in this study included *blocking out distracting stimuli around their homes, staying alert, and reducing cognitive load*.

Participants engaged in routines to *mitigate or block sensory stimuli that would be distracting* in advance of a call they had scheduled. This included sensory stimuli outside of their homes including birds chirping (Connor) or rainstorms (Ian), as well as inside their own homes, such as when another person entered the room where they were videoconferencing (Ian, Connor). Routines we uncovered included putting on headphones in advance (Ian, Connor), closing the door to the room they were in (Ian), and making sure they removed items unrelated to videoconferencing from their desk (Ian). For Ian, blocking distracting sound was particularly important as he had cochlear implants to receive extra sounds. His routine of managing the audio carefully during the videoconference helped him focus on the webinar. He explained that his routine of closing windows was “because you don’t want anything competing for your attention.”

Participants also engaged in routines to *stay alert*, which helped them maintain focus while videoconferencing. For example, Vivian would often be sitting on the couch and turn on the white lights she had installed above the couch. She explained that these lights overrode the dim, distracting yellowish lighting and provided a vibrant and energetic visual cue, helping her stay focused during videoconferences. Other participants relied on caffeinated beverages to maintain an appropriate energy level. Phoebe consistently would prepare coffee and place it next to her computer in advance of any professional videoconferencing session, sipping the coffee throughout a meeting that required her to stay alert. Phoebe engaged in a prayer every day before videoconferencing “to keep her mind ready”.

Participants also developed routines to *lower the cognitive load* involved in engaging in videoconferencing. These types of routines sometimes included making some digital aspects of the videoconference physical, such as when Phoebe would print out materials that would be used in an upcoming videoconference prior to the videoconference, so that she would not need to attend to the information in digital format. By sticking to this method of information processing, she could grasp information easily – Phoebe shared that she would know where to look at, as well as search for information once materials were printed out. Other routines were in response to the cognitive load introduced by the synchronous nature of videoconferencing that could be overwhelming. Both Ian and Connor had developed routines of printing webinar handouts after each videoconference to review and retain important information on their own time (Ian) and taking notes during the videoconference and then reviewing and processing the information afterward (Connor). Participants explained that this removed overwhelm (Ian) or the pressure of immediate recall (Connor). Phoebe, who said her memory used to be more reliable, now wrote down things that would have “come naturally” to her in the past, helping her lower the cognitive load of staying connected to the information during the videoconference. A contrasting strategy occurred with Vivian, who adopted a more relaxed routine in response to memory changes. Her new routine involved choosing not to push herself to recall information when things did not come up immediately during a videoconference, such as the name of a restaurant she wanted to mention but couldn’t remember at the moment. Instead, she allowed the information to surface gradually, letting it come to her in a different time.

### 4.2 Certain Strategies Can Help Manage Disruptions to Routines

Above, we describe different types of routines that supported participants in adapting to different constraints of videoconferencing. Routines, in general and in these instances, are beneficial because they help people respond to disruptions in ways that require little conscious thought [5]**.** At times, however, we saw disruptions *to the routines themselves.* In this section, we describe four strategies that participants employed to **manage disruptions to their routines**: *accepting*, *removing*, and *negotiating* the disruption, as well as *developing new routines* in response to the disruption.

#### 4.2.1 Accepting minor disruptions to routines.

Participants sometimes *tolerated disruptions to their routines* when they did not significantly affect their videoconferencing activity. We observed this when audio froze during videoconferences. For instance, Vivian’s routine involved using the iPad’s built-in speakers to hear the other person’s voice during videoconferences. She mentioned that this routine was disrupted when her brother occasionally faced connection issues during videoconferencing, causing audio disruption. Although this made it harder for her to hear the conversation, she adjusted by guessing what was being said by her brother. While she explained that it was occasionally annoying, she did not feel that it caused serious problems.

#### 4.2.2 Removing disruptions to routines.

Not all distractions were easily overlooked, and participants sometimes responded by *removing* them. For instance, Connor removed lighting that he had added to his home videoconferencing setup but which disrupted a routine. When he had installed ring lights next to his workstation, they made the room brighter and improved his visibility. However, installing the light disrupted his current routine of wearing glasses throughout the videoconference to make sure he would be able to see anything relevant to the videoconference, like text on the screen and letters on the keyboard. This routine was disrupted by the “bouncing off” of the glares and reflections caused by the ring lights. He removed the ring light, concluding that it was a ”failed experiment.”

#### 4.2.3 Negotiating disruptions to routines.

Removing distractions was not always possible or the best approach to managing disruptions. In these cases, participants sometimes *negotiated the disruptions to their routines*. Sometimes this occurred when the distraction was caused by or could be controlled by another person. For example, when there were other people living with participants, it became reasonable to communicate with them about distractions. Connor shared that early in his use of videoconferencing, he had routines to openly communicate with his wife about when an upcoming videoconference would take place. He would also set hints, such as closing the door, to signal that he was in a call, especially when communication had not occurred beforehand. In response, his wife respected his space by intentionally avoiding walking on the same floor while he was in the videoconference. Nowadays, Connor shared they do not need to have those conversations anymore as his wife is aware of his needs during the meeting. Nora, in contrast, continued to notify her husband about when her videoconference was scheduled and for how long so he could avoid interrupting her during the call.

In one instance, the disruption came from other engaged partners in the videoconferences rather than others in the same household. During Nora’s exercise videoconference, the instructor played upbeat music that didn’t align with the calming atmosphere Nora and others needed in order to focus on getting the desired exercise movements. Nora and several others raised this concern with the instructor. This concern was quickly resolved. The instructor played something more suitable for Nora and the others, while wearing headphones to listen to her preferred music that helped her stay energized to lead the exercises.

#### 4.2.4 Developing new routines.

Sometimes, the methods above could not resolve the disruption entirely, or the disruption would recur. In these instances, participants at times *developed new routines* to handle the disruption. After recently moving into a new apartment, Vivian’s new lightning caused distraction due to where it was located. She told us every time she was going to join a videoconference, she would first remove this light distraction by turning off the lights. As another example, Lydia’s sister or other relatives who live nearby often stopped by her home without prior notice, and, in her past experience, were likely to stop by when she was in a videoconference exercise session. To alleviate this disruption, Lydia adopted the routine of always preparing a second set of exercise materials that she kept nearby, so that someone stopping would be able to use this set of exercise gear to do exercise with her rather than requiring that Lydia stop her exercise to provide company to a guest.

### 4.3 Some Disruptions to Routines are More Challenging to Manage

As discussed above, participants employed a number of techniques to manage disruptions to their routines. Some disruptions, however, were more challenging for them to address. We found three situations where **routines faced disruptions that participants were not always able to manage**. This includes when v*ideoconference access routines do not work*, when there are *no alternative ways to reach the communication partner*, and when *new cognitive changes challenge routines* that had previously been effective for participants.

#### 4.3.1 When videoconferencing access routines do not work.

Videoconferencing routines were different depending on participants’ software, devices, and the mechanics of the platform that they used. For example, Vivian’s routine involved clicking on the software embedded as a video feature using a communication app that was widely used where her brother lived. As she described, she would just “open the app and click the video button,” much like using FaceTime. Connor’s access routine was dictated by his company’s working protocol where he followed a set approach: first opening the company software, then licking the link in the e-calendar, making the process clear and routine. Nora and Phoebe had developed a similar access routine that involved storing and syncing all videoconferences into their e-calendar, which included the necessary links.

However, we observed a case where *a videoconferencing access routine was disrupted*, resulting in a barrier that was not manageable. After registering for the event that we were scheduled to observe, Ian received a confirmation email, the only email he received. When he tried to find the access link in his email (his usual routine) six minutes before the start, nothing was there. Ian was frustrated as he tried to find the email in his spam and trash folders. He tried troubleshooting, for example clicking on the website link attached to the confirmation email to navigate to the website page, but did not find any useful information. When he was about to give up, the researcher helped him find a “send link” button on the third-party website to resend the email. By then, forty minutes had passed, and Ian had already missed a significant portion of the webinar. From this frustrating experience, it is possible that Ian will develop a new routine after our study in response.

#### 4.3.2 When there are no alternative routines to reach the communication partner.

Participants had different routines to make sure that they would show up to a videoconference at the right time. Most used emails and calendars to note information about videoconference time and information. For example, Lydia routinely used a physical calendar to make all the videoconferences as well as all of her daily appointments. Nora used an e-calendar to mark events, with a notification system that sent her reminder ten mins before any appointment, and Phoebe had her own calendar journal with her all the time to schedule any videoconference. These were largely software from third parties or crafted or designed by people themselves.

However, when these routines did not work and participants missed a call, or when their videoconferencing partner did not show up, participants faced challenges when they did not have alternative routines of communicating. For instance, Nora’s exercise videoconference had not seemed to require any avenue of communication before the meeting, as the videoconferences were scheduled in a fixed time. When the instructor failed to show up without prior notice, Nora and the other participants waited on the call for over 30 minutes, unsure of what to do.

#### 4.3.3 When new cognitive changes disrupt routines.

As described in some of the prior sections, participants developed routines to adapt to their cognitive concerns. We observed some instances where new cognitive changes disrupted videoconferencing routines in ways that participants were not (yet) able to repair. Phoebe found that her prior routine of going through informational documents a week in advance of a videoconference no longer helped her remember anything the day before the videoconference, and she would have to reread the material again. Connor had the routine of turning his mic on and off to speak up for questions or suggestions, however, he had gradually recognized that he had a hard time remembering if he had muted himself, unmuted himself, or sometimes even worse, when he forgot to log off after these work call. He was half joking that it was “time to retire”.

## 5 DISCUSSION

We studied the routines that older adults with cognitive concerns developed around videoconferencing through observations, interviews, and a modified diary study. For **RQ1**, “How do routines support the use of videoconferencing technology for people with cognitive concerns?”, we found that participants developed routines which helped them navigate videoconferencing constraints. In our study, these routines included staying comfortable, connected, and focused. For **RQ2,** “How do participants respond to the disruption of these routines?”, we found that participants managed disruptions to routines around videoconferencing by accepting them, removing them, negotiating them, or developing new routines in response. We also found three kinds of disruptions to routines that participants were not always able to manage.

Past research has indicated that routines are a key component of everyday life, helping people navigate their everyday life context and associated objects [15, 31, 37]. Studying this topic with older adults responds to calls to critically engage with the everyday context of older adults [11, 48, 62, 68, 72]. Our study is informed by a body of research calling attention to the importance of studying and designing around older people’s routines [15, 31, 37, 63, 71]. This past research has advanced our understanding of how *technologies can support or augment routines.* This includes through generating insights around how to design features of [31] or scaffold [71] the sustained, routine use of a new technology – knowledge which is clearly salient to HCI.

Our study examines the inverse of the focus of this existing research: we study *how routines support technology use*. This area of research is important when we conduct research on technologies that older adults may have already been using for years. Just as studying non-technological objects and routines has become the focus of past work [14], we can study how routines supports technology use - specifically when routines involve technology or when their goals are centered around using technology. Below, we discuss why routines are particularly important to study and support for people with cognitive concerns, the importance of studying older adults’ routines, and methods that can enrich HCI research by uncovering insights into these routines.

### 5.1 Routines to Support Memory and Cognition

Technology researchers working with people with cognitive concerns may particularly benefit from studying and supporting routines. While past work has hinted at the importance of access routines and the role of caregivers in facilitating technology use for individuals with cognitive concerns [49], we lack a comprehensive understanding of the specific, routine practices and challenges associated with technology use of videoconferencing. In addition, prior work has advanced our understanding of how to support people with more advanced cognitive impairment in videoconferencing [91], leaving room to understand the experiences of those with more mild cognitive concerns. Our study provides empirical evidence on how people with cognitive concerns are managing issues in the context of videoconferencing.

Recent work in HCI focuses on making videoconferencing more accessible and adaptable, ensuring that these technologies can support evolving needs [41, 46, 50, 65, 94]. For example, Mittal et al. [46] developed a videoconferencing platform with a visual layout and notification system to facilitate communication in mixed hearing group who experience different levels of hearing loss. Similarly, Nacimiento-García et al. [50] designed automatic captions for video calls to support older adults with visual functional diversity. Designing to support cognitive functions in videoconferencing is more rarely seen. One possible reason is the added complexity posed by cognitive concerns: sustained technology use can exacerbate cognitive strain [9, 89], adding layers of difficulty beyond the challenges already existed. In our data, routines offered two ways to help older adults with cognitive concerns better incorporate technology into their lives: first, **routines helped people regulate and manage the disruptions of everyday lives**. Second, **it helps during navigating progressing cognitive changes.** We discuss this in detail below.

One role of routines particularly relevant for people with cognitive concerns is in supporting focus and minimizing the negative effects of disruptions. While distractions have been documented as challenging for other groups in past work [38], distractions can pose a particular threat to older adults with impaired cognitive processing capacity [21]. Past work has identified distractions, including distracting stimuli, as an issue affecting people with mild to moderate dementia using technology [24]. Our work also shows ways that people are addressing these disruptions.

Another aspect that is important for some participants we worked with is that some are experiencing progressive cognitive changes. To cater to these changes, people engaged in invisible use or work, such as creating physical artifacts like notes, to serve as a reminder to support memory lapse during videoconferences [29]. This work, which adds to cognitive load, is often not taken account in the technology design: examples of these kinds of work include preparation work [34], and work people do after the call [29], like reviewing notes captured from the call. In these cases, we observed routines which had previously worked no longer working. This included Phoebe’s prior routine browsing and memorizing the information indeed for a videoconference a week in advance (Phoebe), and Connor relying on an automatic response (routine) to mute or unmute himself. One area of research that our study points to as important is how to study and support transitions in routines for people experiencing shifts in cognition. This is important for people with dementia, who experience regular progressions in cognitive impairment. The stakes can be high here: Connor is seriously considering retirement due to his progressive cognitive changes and nonfunctional routines. A well-established approach in research and practice is support people experiencing cognitive decline in maintaining their current everyday activities to the maximum extent possible. Routines are the underlying glue to their everyday life activities [69]. **It becomes more apparent that supporting routines that do not work anymore or work less well may support people continually engaging in their everyday activities.** For example, when Connor experienced frustration on not being able to use his old routines to memorize when to mute or unmute himself, videoconferencing should better support these routines by providing subtle, context-aware reminders or feedback. For example, the interface could highlight the mute/unmute button more prominently or use gently auditory cues as reminders.

In this section, we described how routines are particularly important to study with people with cognitive concerns. In our data, routines played roles including but also beyond supporting age-related cognitive concerns. Below, we reflect more broadly on what technology researchers working with older adults can gain from studying routines that support technology use.

### 5.2 Re Importance of Studying Routines Rat Support Technology Use in HCI

Here, we describe five research opportunities (ROs) that studying the routines that support technology use opens for researchers working with older adults in HCI.

#### RO1: Studying routines to surfacing technology needs

Studying routines helps us see some of the “authentic” [1] and “unremarkable” [69] technology that is used, in turn demonstrating some of the needs of participants related to technology use. Studying routines offers an alternative and complementary perspective to what can be learned from other methods such as interviews. For instance, previous research has highlighted how videoconferencing supported older adults’ need to stay socially connected during the COVID-19 pandemic by fostering remote connections and reducing loneliness [60]. Studying routines, then, can help us obtain a fuller understanding of the specific needs and practices involved in technology use. For example, studying routines helped us learn about the need to stay comfortable during videoconference – something we likely would not have found with typical interview questions or even observations (we likely would not have noticed them drinking from a water bottle or connected it to videoconferencing). While something like the need to stay hydrated during videoconferencing may at first seem entirely out of the scope of HCI, Lee et al. [38] noted “being thirsty” as one of the “distracting experiences during videoconferencing” that remote workers experienced. In our context, we argue that these kinds of factors should be understood as accessibility needs – just occurring in the interaction between the environment, technology, and activity rather than solely having to do with the features of a particular interface.

#### RO2: Designing to avoid challenging disruptions to routines

Second, studying routines and surfacing user needs can yield new design opportunities. In our study, design opportunities include to address disruptions when videoconferencing access routines did not work, when there were no alternative ways to reach a communication partner, and when new cognitive changes disrupt routines. Each of these issues merit further research. One example of a research direction that could support situations where videoconferencing access routines that do not work is by researching what kinds of tools, education, or advocacy could support developers in adopting consistent and standardized approaches to delivering access links, such as embedding access details within calendar invites. We can also adopt approaches from past work that provide support to older adults to navigate technology interfaces (e.g.,[20, 33, 95]**)**. Another approach might be to design alternative entry methods (e.g., text messages or QR codes) rather than leaving participants with no other options except dropping out a call.

#### RO3: Gathering empirical knowledge about adaptive routines and avoiding disturbing them

While in the prior paragraph we describe opportunities to address needs we learned about through studying routines, it is essential to note that in many cases, participants have developed routines in response to these needs that are working well. We can learn from these routines. Participants developed routines that address videoconferencing challenges identified in past work, such as “Zoom Fatigue” [6], a state that can arise from nonverbal overload like prolonged eye contact and lack of movement. We observed routines participants employed in response such as Phoebe’s routine of preparing coffee and placing it next to her videoconferencing setup to help her stay focused and energized during professional meetings. A future area of research can uncover the adaptive routines that people develop to respond to challenges (e.g., factors that contribute to poor videoconferencing experiences [38]) surfaced in prior work. In addition to contributing empirical evidence of how people adapt to technology disruptions, our findings around routines has implications for design work. **Re key role of developers and designers, then, would be to ensure that their designs do not get in the way of common routines.** For instance, consider a videoconferencing software that implements automatic camera tracking technology based on detected motion during videoconference. While this feature might aim to ensure participant full presence is always visible during the meeting, it could inadvertently disrupt routines observed in our study, such as following a participant as they lean away from the screen to grab coffee or water. This unintended consequence could make participants self-conscious about their behavior, interfering with routines that previously helped them stay hydrated and engaged.

#### RO4: Designing with routines

Given that new designs may challenge existing routines, another potential role for researchers is to design ***with*** routines. Take Vivian’s case as an example: her brother occasionally faced connection issues during videoconferencing, causing audio disruptions. Vivian coped by guessing what he was saying, finding it mildly annoying but manageable. In situations like this, technology could be designed with routines to be better. For example, real-time audio enhancements could help detect audio disruptions during the call and automatically adjust settings such as boosting volume or providing captions [50]. Future technology could also better integrate users’ routines, embedding them into the design of technology itself. For instance, a system could provide contextual information such as the duration of a videoconference call or expected participants beforehand to help users prepare for their routines (e.g., deciding how much water or coffee to have during the call or what to wear).

#### RO5: Recognizing and supporting older adults as the designers of their technology ecosystems

The social model of disability sheds light on the technology designer’s part in the technology user’s ecosystem. According to the social model of disability (as discussed in HCI e.g., [42]), disability is not located solely within an individual, but is shaped by better or worse environments. This perspective highlights the importance of focusing not only on the individual but also on their specific environment when designing technology. Broad studies of populations may no longer suffice; instead, tailoring support to each individual’s unique environment could lead to more effective outcomes.

In the context of routines, this perspective points to a design opportunity: **individuals should be empowered to become the designers of their own routines within a technology ecosystem.** Researchers and designers, in turn, could take on the role of facilitators, helping users uncover and make sense of their routines. The concept of a technology ecosystem [16] – though from a different discipline – emphasizes the importance of incorporating diverse tools and resources to better understand and support individual routines. We propose a two-step approach to recognize and help people become the designers of their technology ecosystems.

The first step involves supporting people in recognizing and surfacing their own routines. This step is important in its own right: making routines visible can result in benefits such as helping individuals adapt to changes more effectively, as seen in Lee and Dey’s work on pill management systems [37]. Given that routines are often unnoticeable [69], researchers could design features that allow users to log and visualize their technology routines. For example, in our study of videoconferencing, such features might track when and how users access links, set reminders, or prepare for calls. By surfacing this information, users could identify effective routines and pinpoint areas for improvement. In the second step, once routines are surfaced, technologists could derive actionable insights to enhance routines. The researcher should adopt a supportive rather than interventionist role in supporting individuals in designing with routines (as we detailed in RO4). This approach respects users’ autonomy while providing meaningful support for their technology use.

### 5.3 Uncovering Information About Routines

Through our approach which was employed over the course of one week, we were able to uncover situated understandings of participant routines, including how they supported participants in adapting to the constraints of a particular technology, how they developed in response to recurrent disruptions, and where they were vulnerable. Below, we reflect on what we have learned about surfacing routines in the context of past work and offer methodological considerations for others.

Uncovering routines is understood to be difficult. Longitudinal studies, like ethnographies, have been employed in the past to identify and surface routines. Tolmie et al. conducted in situ in vivo observation over the course of a year to uncover and understand the lived details of routines within people’s home environments [69]. This process uncovered nuanced insights, but a year-long ethnography is not always possible. Brereton’s study on *habituated objects* [14] began with a contextual interview, during which the participant highlighted tangible objects of significance in her home. This process led to the identification of items such as a milk jar, a kettle, and a magnifying glass, around which meaningful routines emerged. One routine example around the kettle is building water in the kettle for the morning tea. This revelation of this routine was meaningful in relation to supporting participants’ needs to stay connected with family overseas. Nevertheless, not all routines can be revealed by contextual interviews – many are formed with less conscious awareness or are not even recognized.

Our approach incorporated interviews, observations, and a modified diary study. The observations and interviews complemented each other in the identification and documentation of *objects* associated with videoconferencing routines. These methods also contributed to the creation of the RAO (Routines and Objects) board which provided insights into participants’ videoconferencing *routines*. In completing the RAO board, researchers and participants collaboratively uncovered routines embedded in videoconferencing in their home context [10], helping unveil those routines that were buried in the data collected through observations and interviews. Using the water bottle as an example, we observed the water bottle was placed nearby during their videoconference. Through interviews, we gained more insight into why participants kept the water bottle nearby during the videoconference. When the water bottle was captured on the RAO board, along with other objects like the laptop, keyboard, and mouse, we could find out in the later interview how the routine was integrated with the videoconferencing technology. This routine was surfaced through triangulating data from all three methods of data collection.

Our combination of methodologies also helps in terms of the validity of our analysis. The information that we gathered would be difficult to be justified through observation or interviews alone, as routines involve automatic movements [5] that people are less conscious about and may not think about much and therefore may be difficult to recall. In this way, observation complements interviews well with capturing routines that people were normally aware of until being asked. For example, Vivian’s routine of turning off the lights to avoid distraction during videoconference were captured from the observation. While observations are useful to gather information that is challenging to verbalize, observations alone would be challenging to uncover what is a routine versus a one-off interaction with the environment. Later in the interview, Vivian confirmed that she routinely turned off the lights before videoconference calls.

Our goal with our analysis was not to identify all the routines that exist, but rather to understand how they served participants and how participants respond to disruptions. While we did learn about many routines in order to reach our findings, there are certainly more that we were not able to find due to the duration of our study as well as the limitations of the methods that we employed. Below, we reflect on future opportunities for surfacing routines.

As has been demonstrated in much ubiquitous computing research, sensor-based monitoring technologies can detect and recognize individuals’ activities in home environments, such as walking or moving specific objects, as well as where they take place [37, 47, 66, 96]. However, sensors are unlikely to be successful on their own in adequately capturing routines, for example when routines are developed based on other members of the household, such as routines of negotiating with other family members to ensure a quiet space to videoconference. Thus, there are opportunities to utilize sensors as additional means of uncovering information that could then be brought up in interviews. They offer a precision that does not rely on participant recall or researcher direct observation. For instance, in cases where routines involve physical movements like closing the door, sensors can capture this data to contextualize during subsequent interviews if people and researchers are not aware of this routine. Information from sensors might also be more easily used than qualitative observations to support AI-driven research on prompting systems [23, 97] and automating research [40]. However, risks such as privacy concerns [52], potential overreliance on automated data, and reduced participant agency need to be carefully considered here.

Another approach to surfacing routines is to take an **object-focused** view. Uncovering routines is usually coupled with surfacing objects associated with the routines: in this work, we learned about routines by identifying objects that participants captured through the modified diary study. Moreover, we found that even when routines belong to the same category (e.g., maintaining a comfortable body position), the objects embedded in these routines varied depending on the objects available in participants’ surrounding environments (e.g., Vivian’s selection of a couch and Ian’s selection of a comfortable chair). Previous work also went through an *object-identification* journey for identifying routines such as the door in Tolmie et al.’s work [69], and the water kettle in Brereton et al. [15]. Objects hint at the ways that people are entangled with the material word. Specifically to enable technology in use [29], objects-focused view keep a promising future to empower older adults autonomy and interdependence [48, 62] by exploring the tools and resources people correlate„ and understand how future technology might arrive and make its place appropriately in older adults’ day-to-day lives.

## 6 Conclusion

This paper studied existing routines that older adults with cognitive concerns have developed around videoconferencing technologies, revealing how these routines help maintain comfort, connection, and focus during videoconferences. As videoconferencing becomes more embedded in daily life, understanding these routines provides valuable insight into how people incorporate technology into their everyday lives. Our analysis further revealed how they managed disruptions to videoconferencing and videoconferencing routines, and the kinds of routines that were more vulnerable to disruption. We discussed why routines are particularly important to study and support for people with cognitive concerns, the importance of studying older adults’ routines for supporting technology use, and methods that can enrich HCI research by uncovering insights into these routines. Future research should continue exploring how routines evolve over time for informing more meaningful technology design for older adults, including those with memory and other cognitive concerns.

## Figures and Tables

**Table 1:** Participant Demographics.

Pseudonyms	Age	Race/Ethnicity	Memory or Other Cognitive Concerns	Number of Year Experienced	Examples
Vivian	70s	Asian	Subjective cognitive decline	2	Experienced difficulty remembering items placement.
Ian	80s	White	Subjective cognitive decline	1	Experienced sudden spatial confusion - occasionally lost sense of where he was.
Connor	60s	White	Subjective cognitive decline	2	Experienced difficulty finding the exact phrases or words; occasionally did not complete daily tasks (e.g., left the door or a drawer open, wrote emails but forgot to send them, lost focus).
Lydia	80s	Asian	Subjective cognitive decline	2	Found it difficult to remember names and numbers; occasionally forgot to bring important items (such as personal checks).
Nora	70s	Asian	Subjective cognitive decline	2	Experienced challenges with spatial navigation and recalling specific routes; experienced difficulty remembering items placement.
Phoebe	70s	White	Mild cognitive impairment	10	Experienced difficulty remembering names; experienced sudden spatial confusion; experienced difficulty retaining information that was read.

## CCS Concepts

• Human-centered computing; • Human computer interaction (HCI); • Social and professional topics; • User characteristics; • Age; • Seniors; • People with disabilities;

## Footnotes

1 Subjective cognitive decline (SCD) refers to an individual’s perception of a decline in memory or other cognitive abilities compared to their prior performance, despite no measurable neuropsychological deficits being present [55].

2 Mild cognitive impairment (MCI) describes an early phase of cognitive decline, such as memory, language, or spatial ability loss, where individuals can still perform most daily tasks independently [80].

3 Dementia encompasses a group of conditions that impair memory, thinking, and the capacity to carry out daily activities [90].

4 For Mandarin data, the original language was used for coding and subsequent analysis procedures. Translated quotes and data were used for final publication [77].

5 While it is possible to move to different rooms while on a video call, we did not see this often. All participants remained in a fixed location for the entire duration of the calls we observed.
